# Effects of educational intervention based on PRECEDE model on self care behaviors and control in patients with type 2 diabetes in 2012

**DOI:** 10.1186/2251-6581-13-72

**Published:** 2014-07-16

**Authors:** Mahboobeh Borhani Dizaji, Mohammad Hosein Taghdisi, Mahnaz Solhi, Seyed Mojtaba Hoseini, Zahra Shafieyan, Mostafa Qorbani, Morteza Mansourian, Abdurrahman Charkazi, Aziz Rezapoor

**Affiliations:** 1 PhD student in health education and promotion, Tehran University of Medical Sciences, Tehran, Iran; 2 Associate Professor, Department of Health Education and Health promotion, Iran University of Medical Sciences, Tehran, Iran; 3 PhD student in Exercise Physiology, Mazandaran University of Physical Education and Sport Sciences, Babolsar, Iran; 4 DVM; Hospital management research center, Iran University of Medical Sciences, Tehran, Iran; 5 Assistant Professor, Department of Public Health, Alborz University of Medical Sciences, Karaj, Iran and Non-Communicable Diseases Research Center, Endocrinology and Metabolism Research Institute, Tehran University of Medical Sciences, Tehran, Iran; 6 Assistant Professor, public Health department, Ilam University of Medical Sciences, Ilam, Iran; 7 Assistant Professor, public Health department, Golestan University of Medical Sciences, Gorgan, Iran; 8 Assistant Professor, Department of Economic Health, school of health management and information sciences and Health management and economic research center, Iran University of Medical Sciences, Tehran, Iran

**Keywords:** Educational intervention, PRECEDE Model, Self-care, Type 2 diabetes

## Abstract

**Background:**

Diabetes is a chronic disease and its control requires essential change in patients' life style. The aim of this study was survey of effects of educational intervention based on PRECEDE Model on self care behaviors and control in patients with type 2 diabetes.

**Methods:**

This was a quasi-experimental study carried out in 78 patients with type 2 diabetes who have referred to Minoodasht clinic of diabetes. The educational program has been designed according to the PRECEDE Model. Prior to perform the educational intervention, the patients filled a questionnaire which was designed according to the structure of PRECEDE Model for type 2 diabetes patients. The diabetes education program was performed on three target groups (patients, their families and Health care personnel). After four weeks, the effects of the educational program have been evaluated through the same questionnaire. The findings were analyzed by SPSS version 16 and p-value less than 0.05 was taken as statistically significant.

**Results:**

The mean age of participants was 49 years, 87.2% were married and 19.2% was illiterate. The rate of income of 44.9% was low. 66% had a family history of diabetes and 64% had been afflicted with diabetes more than 5 years. The Chi-square test showed a significant relationship between formation of a file in diabetes clinic and on-time presence to receive services and participation in the educational classes with the marital status variable. The results also showed that there is a significant relationship between observing food diet and job. The mean scores of knowledge, attitude, practice, reinforcing factors and enabling factors has increased after educational intervention. The Chi-square test shows a significant difference before and after of education intervention in stages of the model.

**Conclusion:**

The obtained results based on PRECEDE Model would support the positive effect of the educational intervention and its major elements (predisposing, enabling and reinforcing factors) on diabetes self-care behaviors.

## Introduction

Diabetes is a prevalent disease which can lead to metabolism disorders, health problems and chronic consequences including diseases of kidney, heart, blood vessels, vision and etc., which are created both as a result of genetics and environmental behavioral factors [1].

In recent decades, the number of individuals afflicted with diabetes is increasing rapidly, the spread of diabetes of type 2 will increase from 171 millions people in 2000 to 366 millions people in 2030 [2]. In general, the results related to outcomes and control of diabetes has proved that a well metabolic control and sufficient education can improve the clinical outcome of people with diabetes [3].

According to WHO, education is the base and infrastructure of diabetes treatment. The key purpose of diabetes education is to change the behavior of individuals and promotion of self-care [4]. In recent decades, various studies have shown that education of patients is accompanied with the reduction of chronic consequence of diabetes [5].

The models of planning provide a framework for the health trainers to produce a program. At present, one the most famous and applied applicable theory is The PRECEDE^a^ model. In a first look, The PRECEDE model seems to be very complex, but having it examined closely, it will be learned that there is a very logic sequence in the 9 mentioned phases and will cause the design of the planning process for the health promotion. The PROCEED model starts with end results and moves in the back direction to reach the causes [6].

This model shows that how the social diagnosis, epidemiology and behavior lead to a clear understanding of the society individuals’ needs, problems and desires. It also examines those groups of behavioral factors which have a close link with health and specifies the pre-behaviors or factors affecting the behavior in educational diagnosis such as predisposing factors (knowledge, attitude, etc.), reinforcing factors (impacts of others, family, peer groups, etc.) and enabling factors (accessibility of resources, skills, etc.) [7,8].

Unfortunately, the education of patient is given less importance as compared with other clinical actions and in most cases [9]. It should be taken into consideration that the use of theory will increase the effect of educational program and help with the identification of individuals’ features and their surrounding environments which have impact on behaviors in one way or another [10].

Considering the fact that the promotion of self-care behaviors by diabetic patients are under the influence of different sources and any kind of action to its increase should be made with regard to individual, environmental and social factors, also taking into consideration that the framework of PERCEED model examines effective individual and environmental factors on a problem, so the framework of this comprehensive model was used to promote the diabetic patients’ self-care behaviors.

The effective application of this model has been proved in various health subjects. For instance, Jalili could show that educational programs based on The PRECEDE model has had a greater effects in the correction of mothers’ nutritional behaviors and their children’ blood indexes as compared with classic education and prescription of supplementary iron [11]. Also, the framework of PRECEDE model was used to diagnosis the obstacles of screening the women’s Cervix cancer [12].

This research has been conducted in Iran due to the increase of the statistics of diabetes and the lack of a written educational plan based on educational and behavioral models and theories with an aim to plan based on PRECEDE model and its impact on the promotion of self-care behaviors of the patients with diabetes type 2 to improve their quality of life.

## Methods

This study was performed through following stages:

At the stage of social diagnosis, the patients’ quality of life was determined based on the questionnaires of WHO. Moreover, it was conducted based on the data collected from the quality of life questionnaire and previous researches on this area, primary feasibility studies to intervene based on the identification of patients. Afterwards, the social problems which had a negative impact on the diabetic patients’ quality of life were specified.

At the stage of epidemiologic diagnosis, the patients’ health problems were determined with regard to a review of the texts and findings of the interview with patients and also the primary studies made by the researcher on patients (lack of self-care skills). At this stage, the general goals and exclusive objectives of educational program were compiled.

Reviewing the texts and findings of the interview with patients, the lack of self-care skill included: the lack of control of blood sugar, blood fat, blood pressure and weight through inappropriate nutritional diet and the lack of physical activities, sport and quit of cigarette smoking.

The variables of age, gender, income, academic level, job, body mass index, (BMI) duration of affliction with disease and genetics were considered as the non-health factors affecting the diabetic patients quality of life.

At the stage of behavioral diagnosis, the behaviors classification has been made on the basis of importance. Finally, certain behaviors, such as consumption of prescribed medicine by physician, control of blood sugar, fats, blood pressure and observing food diet, exercise, formation of file in diabetes clinic and on-time presence in educational classes, were determined as behavioral factors affecting health problems. Also, age, gender, genetic potential and lack of facilities to do exercise, lack of observing food diet and lack of free-of-charge facilities to measure blood sugar, fat and blood pressure were determined as effective non-behavioral factors.

At the stage of educational diagnosis, the predisposing, enabling and reinforcing factors were reviewed. Reviewing the primary studies by the researcher, the knowledge, attitude, belief and values were selected as predisposing factors to conduct self-care behaviors. The enabling factors of self-care behaviors included sufficient skill to control diseases of (sugar diabetes, blood pressure–weight) and having resources, educational classes and family support. The reinforcing factors including the positive experiences of patients, family encourage and involved staffs were determined in the research.

With regard to these stages, the standard and self-made questionnaires were prepared. After preparing the related questionnaire, 78 patients referring to the Diabetes Clinic of Minioodasht City (Golestan province) were studied. The sample volume with regard to the conducted research in promotion of self-care behaviors in diabetic patients was with 95% reliability and the power of test of 80% and forecasting a ten percent fall of the sample were estimated to be at least 78 people.

In this research, the tools to collect data included a questionnaire developed in three parts. The first part of this questionnaire included questions related to demographic factors including age, gender, job, level of education, marital status, number of children, rate of monthly income, duration of affliction with diabetes, body mass index, and the record of the family of this disease and genetics.

The second part was the checklist of behavioral causes affecting the disease. This checklist included 7 questions (drug use as directed by physician, control of blood sugar, blood pressure and blood fat, exercise, having a file in diabetes clinic and pervious participation in educational program.

The third part of the questions included the predisposing factors (knowledge, attitudes, beliefs and values), enabling factors and reinforcing factors.

The knowledge questions within the format of 8 closed questions, attitudes with 14 questions in Likert scale (Agree Strongly, Agree Moderately, no comment, Disagree Slightly, Disagree Strongly), enabling factors within the format of 9 questions (Available resources and facilities, educational programs, family support and skills), and reinforcing factors within the framework of 3 questions (Positive experience of patients, families support and staff support) were designed respectively.

As for the key to the question, scores were given in the following way: The correct responses to the questions were given one score and the negative responses to questions were not given any scores. In order to validate the self-made questionnaire, the method of content credit was used. The questionnaire was submitted to 10 related professors and their corrective views were applied.

In order to calculate the reliability of these questionnaires, the method of calculation of re-exam with a two-week time span for 15 patients was used. The obtained correlation for the re-exam from the self-made questionnaire was r = 0/8.

By the way, the questionnaires were completed in form of interview by researcher. Having completed the questionnaires, the data were analyzed. Then, the educational objectives and contents were adjusted in three recognition, attitude and behavioral areas and the educational materials were selected.

The educational intervention was adjusted in 5 weeks and one session was performed in each week. The duration of each session was flexible between 20 to 30 minutes with regard to the patients and their conditions. The educational CD, pamphlet, tract and brochures were prepared on diabetes, nutrition, sport and false beliefs and presented to patients. Also, lecture and question and answer, group discussions and broadcasting educational films were used to educate the patients.

In the meetings of reference, the patients were given their own special diet program by the nutrition expert. As for the type and method of doing useful sport activities of diabetic patients, they underwent practical exercises in a group form.

In educational meetings, it was decided to have one member of their family present. An educational pamphlet was prepared to increase the support of patient family and helping with him/her in controlling the diabetes. In order to increase the support of personnel in respect of diabetic patients, an educational meeting was held for the personnel. Meanwhile, the positive experiences of patients were considered to be a reinforcing resource. Finally, four weeks after the end of intervention, the impact of educational program on the promotion of patients’ self-care behaviors was measured.

Using SPSS statistical software, the collected data were analyzed. In order to determine the existence of a significant difference among the scores of different dimensions of life quality and disposing, enabling and reinforcing factors in the pre-stage and four weeks after educational intervention in patients, the statistical paired t-test was employed.

This research has been approved by the Research Ethics Committee of Iran University of Medical Sciences: by ethical code P/74

## Results

The mean age of participants was 49 years, 79.5% were women and 20.5% were men. 87.2% were married and 12.8% were widow. The greatest percent of units under research (76.9 percent), 19.2% of the participants under consideration were illiterate, 43.6%with an education at the level of primary school, 5/1% at the level of guidance schools, 7.7% at the level of high school and 24.4% with a high school and diploma but none of them had a university education. 17.9% of the participants had 2 children or they were with less than 2 children, 38.5% with 3–5 children, and 43. 6% with more than 5 children. The rate of income of the greatest percentage of participants (44.9 percent) low, 33.3% an average income and 21.8% high income have been reported. 55.1% had overweight, 7.7% had an obesity of degree 1. Almost 66% of individuals had a family history of diabetes disease and 64% of the whole sample had been afflicted with this disease more than 5 years. 10.3% Two years or less had a history of diabetes, 25.6% had a 3–5 years history of diabetes and the rest more than five years had history of diabetes.

The Chi-square test showed a significant relationship between formation of a file in diabetes clinic and on-time presence to receive services and participation in the educational classes with the marital status variable. In other case, there is no significant relationship between self-care behaviors (observing special diet, necessary test to control blood sugar with a physician’s view and doing exercise) and the marriage status.

The Chi-square test also showed that there is a significant relationship between observing food diet and job and there was no significant relationship between self-care behaviors and job. The results showed there is a significant relationship between behaviors such as observing nutrition diets, doing exercise and formation of file in diabetes clinic and receiving services, on time presence in educational classes in clinic and the variable of educational level. The distribution of the absolute and relative frequency of the behavior before and after educational intervention shows in Table 1.

**Table 1:** The distribution of the absolute and relative frequency of the behavior before and after educational intervention in research units

**McNemar statistical test p-value**	**Post- intervention**		**Pre- intervention**		**Behavior**	
	**Percent**	**Number**	**Percent**	**Number**		
p < 0/001	94.9*%*	74	69.2*%*	54	appropriate	Observing special diet
	55.1*%*	4	30.8*%*	24	inappropriate	
p < 0/004	74.4*%*	58	62.8*%*	49	appropriate	carrying out necessary tests for controlling blood sugar based on the physician opinion
	25.6*%*	20	37.2*%*	29	inappropriate	
p < 0/001	62.8*%*	49	35.9*%*	28	appropriate	playing sport
	37.2*%*	29	64.1*%*	50	inappropriate	
p < 0/001	98.7*%*	77	97.4*%*	76	appropriate	Establishing a file in diabetic clinic
	1.3*%*	1	2.6*%*	2	inappropriate	
p < 0/001	52.6*%*	41	33.3*%*	26	appropriate	On time referring to get the services and participating in clinical training courses
	47.4*%*	37	66.7*%*	52	inappropriate	

The results show a significant relationship between receiving services on time and participation in educational classes in clinic with the income level.

The Chi-square test does not show a significant relationship between the behavior and body mass index (BMI) and between the scores of knowledge, attitude, behavior, enabling factors, and reinforcing factors before and after intervention, there is no significant relationship. The mean scores of knowledge, attitude, behavior, reinforcing factors, and enabling factors has increased after educational intervention. The Chi-square test shows a significant difference before and after of educational intervention in stages of the model (p < 0/001) (Table 2).

**Table 2:** A comparison of the mean and standard deviation of the scores of knowledge, attitude and behavior, enabling factors and reinforcing factors before and after intervention

**Paired-t-test**		**Post- intervention**		**Pre- intervention**		**Studied variation**
**t**	**p- value**	**Standard deviation**	**Mean**	**Standard deviation**	**Mean**	
−8/93	p < 0/001	2.41	15.32	4.04	12.66	knowledge
−8/20	p < 0/001	5.15	56.15	5.42	54.01	Attitude
−6/16	p < 0/001	0.94	5.29	1.09	4.64	Enabling factors
−6/65	p < 0/001	0.63	2.41	0.8	1.88	Reinforcing factors
−7/94	p < 0/001	0.87	4.83	1.02	3.99	Behavior

The results did not show any significant relationship between self-care behaviors (observing specific nutritional diet, conducting necessary tests to control blood sugar with regard to the physician views, performing exercise, forming a file in a diabetic clinic and on-time presence to receive services and participation in educational classes) and the gender variable.

So, the above results confirm the positive impact of the program of educational intervention based on PRECEDE model and its main components (disposing factors, enabling factors and reinforcing factors) on behaviors controlling diabetes (Table 2).

## Discussion

Based on the results of this research, the mean score of knowledge after educational intervention has increased. This increase indicates the positive impact of education in changing the patients’ knowledge. These findings are compatible with the results of related and similar studies [13-22]. Another main variable under consideration in this research was the attitude of the patients. Reviewing the score difference of individuals’ attitudes before and after educational intervention, a significant difference was observed (P < 0/001). This finding is fully similar to the results of studies conducted by Rakhshandeh Roo et al., Rezaei et al. and Heidari et al. Of course, it must be said that change in attitude is one of the difficult stages of educational interventions. All studies which achieve a significant change in knowledge are not necessarily successful in changing the attitudes too.

For example, Rakhshandeh Roo in a research by Shabbidar et al., entitled, “The impact of education of nutrition on the level of knowledge, attitude and practice of diabetic patients type 2”: A comparison of individuals’ attitudes towards diet and health” showed that there was no specific difference between intervention group and standard group after 3 months of intervention and 3 months follow up [17]. In this research, the results showed that the mean score of behavior has increased after educational intervention. (p < 0/001) and the educated individuals had a better practice as compared with diabetes disease. In this study, only there were 26 (33.3%) people who had referred to clinic before education to receive services and participate in educational classes on time.

This number increased by 41 (52.6%) after education. With regard to the results of present research and studied conducted by Larijani et al., participation in group meetings has a better impact on knowledge and practice of the patients under consideration [23]. According to the Australian researchers, these classes should be held constantly in regular time span [24]. Findings in this research showed that 69.2% of individuals were observing a specific diet and this number increased by 94.9% after education.

Diabetes is among the diseases whose main part of treatment is undertaken by patients and their enjoyment of knowledge in different areas of treatment in particular nutrition management is of high importance. In a study by Sharifi Rad et al., after educational intervention, the score of nutritional practice of patients in the intervention group had a significant increase which is compatible with the results of this study [25].

In another study by Sun et al., it was specified that the components of PRECEDE-PROCEED model in nutritional education in line with change in nutritional behaviors of Chinese-American students are very effective [26]. The results of this study showed that the number of individuals who had done necessary tests to control blood sugar before education based on physician’s view were 49 people (62.8%) and this number increased by 58 (74.4%) after education. 28 (35.9%) were doing sport exercise before education, and this number reached by 49(62.8%) after education.

A study which was conducted by Agha Molayee et al. is compatible with the results of this study. In this research, there was a significant increase in the mean of knowledge, physical health, mental health and a significant reduction in the average of Hemoglobin A1c and also a significant increase from the viewpoint of the personal control of blood sugar, weight control, sport and observing diet [27].

A study by Asgari Sari et al., in Kashan also showed that the lack of sufficient education and orientation of patients in respect of nutrition diet, proper consumption of medicine and environmental conditions have impacts on the high spread of diabetes [28].

The findings of this research are in agreement with the research of other studies in which the framework of PRECEDE-PROCEED model has been used for educational planning. For example, Shakoori in a research entitled, “The effect of educational plan of health based on PRECEDE model on the control of Anemia of the deficiency of iron in female students of high schools, in Talesh city showed that the program of educational intervention based on PRECEDE model and its main components (disposing, enabling and reinforcing factors) have had a positive impact on the increase of preventive behaviors of iron deficiency anemia in the society under consideration [29].

Appropriate education is one of the basic points in the promotion of knowledge, attitude and practice [16]. The results of this study showed that design and implementation of educational program in accordance with The PRECEDE model can create a significant difference in the rate of knowledge, attitude, enabling and reinforcing factors of patients before and after intervention and conducting effective self –care behaviors in patient.

The trend of prevalence of diabetes has highlighted the importance of this disease as a public health problem. Different studies have shown that knowledge, attitude and practice of diabetic patients towards diabetes are effective in the control of disease and reduction of its consequences [28].

## Conclusion

Since education is one of the main pillars of health and treatment cares and part of patients’ rights, it is necessary for our country to pay more attention to educational design and planning based on educational models, theories and behavioral & social sciences for different health diseases and subjects. The PRECEDE model is considered to be a comprehensive model in planning for the education of health and promotion of it and it can be used for different health subjects.

### Limitation of study

1 -we had some limitation for traveling the patients to diabetes clinic and providing educational materials.

2 -Some patients were not desire to participate in the study, we explained the importance of the study for them and competition were held in health issues and prizes were given to the winners.

## Consent

Informed consent was obtained from all patients for the publication of this report and any accompanying images.

## Endnote

^a^Any substance to which subjects were sensitive and had mentioned it in the questionnaire.

## Abbreviations

PRECEDE: Predisposing, Reinforcing and Enabling Constructs in Educational Diagnosis and Evaluation.

## Competing interests

There are not any financial or non-financial competing interests (political, Personal, religious, ideological, academic, intellectual, commercial or any Other) to declare in relation to this manuscript.

## Authors’ contribution

MBD, MHT and MS participated in study designed and data acquisition. SMH, MM and ZS participated in statistical analysis and interpretation of results. MM and MQ participated in statistical analysis and drafted the manuscript. AC and AR participated in drafted the manuscript. All authors read and approved the final manuscript.

## References

[B1] Tuomilehto J Lindström J Eriksson J Valle T Finnish Diabetes Prevention Study Group. Prevention of type 2 diabetes mellitus by changes in lifestyle among subjects with impaired glucose tolerance N Engl J Med 2001 344 18 1350 1343 10.1056/NEJM200105033441801 11333990

[B2] Meneilly GS Tessier D Diabetes in elderly adults J Gerontol Series Biol Sci Med Sci 2001 56 1 5 13 10.1093/gerona/56.1.M5 11193234

[B3] Rakhshanderou S Heydarian AR Rajab A The Effect of Health Education on Quality of Life in Diabetic Patients Referring to Iran Diabetes Association Daneshvar J 2006 13 63 15 20

[B4] International Diabetes Federation (IDF) Diabetes Atlas. Prevalence estimates of diabetes 70–20 years 2007 [(adjusted to world population). Available from: URL: http://www.eatlas.idf.org/atlasffd.html]

[B5] Mollaoglu M Beyazıt E Influence of diabetic education on patient metabolic control Appl Nurs Res 2009 22 3 190 183 10.1016/j.apnr.2007.12.003 19616166

[B6] Safari M Shojaeizadeh D Theories Models and methods of health education and promotion 2009 1 Asar-e Sobhan publishing 22 24

[B7] Green L Kreuter M Health promotion planning: An educational and environmental approach 1991 2 London: Mayfield Publishing Co 478 507

[B8] Rahman S A nutritional profile of non-pregnant women from the slums of Dinajpur Bangladesh Trop Dr 1999 29 4 221 224 10.1177/004947559902900410 10578636

[B9] Deyirmenjian M Karam N Salameh P Preoperative patient education for open-heart patients A source of anxiety Patient Educ Counsel 2006 62 111 117 10.1016/j.pec.2005.06.014 16530377

[B10] Jackson C Behavioral science theory and principles for practice in health education Health Educ Res 1997 12 2 143 150

[B11] Jalili Z Application of the educational PRECEDE model to do a causative analyze of preventive behaviors of mothers in iron deficiency anemia of 1–5 year children J Kerman Univ Med Sci 2010 9 2 93 102

[B12] Hislop TG Deschamps M Teh C Jackson C Tu SP Yasui Y Schwartz SM Kuniyuki A Taylor V Facilitators and barriers to cervical cancer screening among Chinese Canadian women J Public Health 2003 94 1 73 68 10.1007/BF03405056 PMC6979973 12583683

[B13] Hawthorne K Effect of culturally appropriate health education on glycaemic control and knowledge of diabetes in British Pakistani women with type 2 diabetes mellitus Health Educ Res J 2001 16 3 381 373 10.1093/her/16.3.373 11497119

[B14] Hazavehei M Khani Jyhouni A Hasanzadeh A Sahrifi M The effect of educational program based on BASNEF model on diabetic (Type II) eyes care in Kazemi's clinic,(Shiraz) Iran J Endocrinol Metab 2008 10 2 154 145

[B15] Rezaei N Tahbaz F Kimyagar M Alavi Majd H The effect of nutrition education on knowledge, attitude and practice of type 1 diabetic patients from Aligoodarz J Shahrekord Univ Med Sci 2006 8 2 52 59

[B16] Aghamolaei T Eftekhar Ardebili H Effects of a health education program on behavior, hbA1c and health related quality of life in diabetic patients Acta Medica Iranica 2005 43 2 94 89

[B17] Shabbidar S Fathi B Effects of nutrition education on knowledge and attitudes of type 2 diabetic patients J Birjand Univ Med Sci 2007 1 14 31 36

[B18] Heidari GH Moslemi S Montazerifar F Haghanifar S The effect of nutrition education on the knowledge, attitude and practice of type 2 diabetic patients Tabibe Shargh 2002 4 4 207 213

[B19] Ghazanfari Z Ghofranipour F Tavafian SS Asadolah R Ahmadi F Lifestyle education and diabetes mellitus type 2: a non-randomized control trial Iran J Public Health 2007 36 2 68 72

[B20] Milenkovic T Gavrilovic S Percan V Petroveski G Influence of diabetic education on patient well-being and metabolic control Diabetol Croat 2004 33 3 95 91

[B21] Sharifirad G Kamran A Entezari MH Effect of dietary regulation education on fast blood glucose and body mass index of type 2 diabetic patients J Ardebil Univ Med Sci 2007 7 4 380 375

[B22] Tankova T Dakovaska G Koev D Education of diabetic patients a one year experience Patient Educ Couns 2001 43 2 145 139 10.1016/s0738-3991(00)00159-2 11369147

[B23] Larijani B Fakhrzadeh H **A comparative study of the impact of education through holding group meetings and educational booklets on the rate of** knowledge **and practice among the patients referring to Lipid Clinic** J Diab Lipid Iran 2003 3 1 89 98

[B24] Tyszecka G Analysis of reasons for discontinuation of treatment for dyslipidemia in secondary prevention of coronary heart disease Przegla lekarski 1995 56 11 709 716 10800583

[B25] Sharifirad GH Entezari MH Kamran A Azadbakht L The effectiveness of education of nutrition on diabetic patients type 2: The application of the model of health belief J Diab Lipid Iran 2008 7 4 379 386

[B26] Sun W Sangweni B Effects of a community-based nutrition education program on the dietary behavior of Chinese-American College students Health Educ Int 1999 14 3 241 249

[B27] Agha Molayee T Eftekhar H Application of the model of health belief in changing the behavior of diabetic patients Payesh J 2005 4 4 263 269

[B28] Asgari SM Alavi S A study of the causes for the prevalence of diabetes in some rural regions of Kashan city J Tehran Univ Med Sci 1995 12 2 80 89

[B29] Shakoori S Sharifi Rad G The impact of educational program of healthy based on PRECEDE model on the control of iron deficiency anemia in the female students at high schools of Talesh city Arak Univ Med Sci J 2009 3 48 57

